# Deciphering the Metabolome under Stress: Insights from Rodent Models

**DOI:** 10.2174/1570159X21666230713094843

**Published:** 2023-07-13

**Authors:** Maria P. Papageorgiou, Daniela Theodoridou, Markus Nussbaumer, Maria Syrrou, Michaela D. Filiou

**Affiliations:** 1 Laboratory of Biochemistry, Department of Biological Applications and Technology, School of Health Sciences, University of Ioannina, Greece;; 2 Biomedical Research Institute, Foundation for Research and Technology-Hellas (BRI-FORTH), Ioannina, Greece;; 3 Laboratory of Biology, Faculty of Medicine, School of Health Sciences, University of Ioannina, Greece;; 4 Ιnstitute of Biosciences, University of Ioannina, Greece

**Keywords:** Metabolomics, acute stress, chronic stress, early life stress, restraint stress, social defeat, mass spectrometry, mice

## Abstract

Despite intensive research efforts to understand the molecular underpinnings of psychological stress and stress responses, the underlying molecular mechanisms remain largely elusive. Towards this direction, a plethora of stress rodent models have been established to investigate the effects of exposure to different stressors. To decipher affected molecular pathways in a holistic manner in these models, metabolomics approaches addressing altered, small molecule signatures upon stress exposure in a high-throughput, quantitative manner provide insightful information on stress-induced systemic changes in the brain. In this review, we discuss stress models in mice and rats, followed by mass spectrometry (MS) and nuclear magnetic resonance (NMR) metabolomics studies. We particularly focus on acute, chronic and early life stress paradigms, highlight how stress is assessed at the behavioral and molecular levels and focus on metabolomic outcomes in the brain and peripheral material such as plasma and serum. We then comment on common metabolomics patterns across different stress models and underline the need for unbiased -omics methodologies and follow-up studies of metabolomics outcomes to disentangle the complex pathobiology of stress and pertinent psychopathologies.

## 1 INTRODUCTION

### 1.1 The Burden of Stress in Modern Societies

Our modern, fast-paced way of life is stressful. Stress refers to the response to a challenging stimulus and should not be interchangeably used with anxiety, which is a state of uneasiness and enhanced vigilance in the absence of an immediate threat [1]. Psychological stress contributes to disease burden in all chronic conditions, and chronic stress is one of the major risk factors for psychiatric disorders [2]. Spiritual approaches [3], as well as meditation [4] and yoga practices [5], have been found to exert beneficial effects as stress-coping strategies in modern societies. Clinical studies underline that repeated stress causes structural and functional changes in brain regions involved in emotional processing, such as the hippocampus, prefrontal cortex and amygdala, which are also implicated in the pathophysiology of depression [6, 7]. The duration and intensity of the stressor, the developmental time point of stress exposure, the unique interplay of the genetic make-up and the environment for each organism as well as confounding pathologies and diverse lifestyles shape the heterogeneity and the multifactorial nature of stress responses in humans.

## 2 HOW TO MODEL STRESS IN RODENTS?

**Fig. (1):**
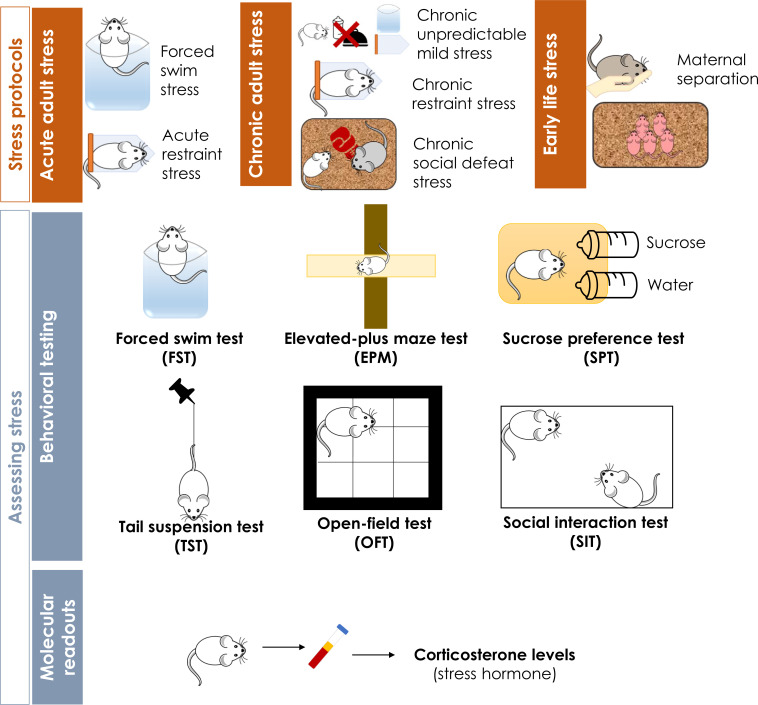
Common acute, chronic and early life stress protocols used in rodents along with behavioral and molecular tests to evaluate stress exposure effects. Only behavioral tests used in the metabolomics studies discussed are presented.

Preclinical animal models of stress, namely mice and rats, provide valuable insights into understanding stress responses in a way that is more comprehensive to study and facilitate investigation of stress effects in a controlled environment [8]. Studying rodent stress models is also largely beneficial due to the conservation of brain circuits that orchestrate emotional processing in rodents and humans, such as fear or anxiety [9], allowing additional ethological comparisons. A variety of protocols have been established to induce stress in rodents which can be classified according to their duration as acute or chronic. Stress can be applied to adult animals or during a rodent’s early life, from parturition until the age of weaning [10, 11]. Here, we review some of the most routinely used protocols for acute (adult), chronic (adult) and early life stress in rodents for which metabolomics studies have been performed (Fig. **1**).

### 2.1 Acute Stress (Adult)

#### 2.1.1 Acute Restraint Stress (ARS)

Restraint stress is a routinely implemented paradigm to induce stress in rodents which is easy to carry out, painless and does not cause lasting physical damage to the animal. Additionally, the parameters of the procedure can be changed reliably and precisely. The subject is placed in a container which restricts locomotion to some degree. Different types of containers have been used, including (but not limited to) plexiglas tubes, decapicones and wire mesh containers. Acute restraint stress (ARS) is used as a paradigm which combines physiological, psychological, and intense stress stimulus and produces emotional and autonomic responses. ARS duration can vary from several minutes up to several hours and when repeated, it can be used as a chronic stressor (see ‘Repeated restraint stress (RRS)’). Restraint may occur under bright light, ambient light or dark conditions. Effects of restraint stress on physiology can be measured reliably in the brain and blood [12].

#### 2.1.2 Forced Swim Test (FST)

During forced swim stress (FST), the animal is placed in a beaker containing water (typically 22-25°C) from which it cannot escape. Over time, the animal displays episodes of immobility, during which all movements, except for those necessary to keep its head above water, cease. Treatment with antidepressant compounds alters the duration and onset of this behavior (see also *3.2.3*) [13]. Apart from the screening of antidepressants, an increasing body of literature also highlights the FST as a stressor and the animal’s reaction as stress-coping behavior [14]. For mice, the typical duration is 5-6 min in a single test session, while for rats, durations of 10-15 min in the pretest and 5 min in the test session have been reported [15]. After the end of the session, the animal is dried with a towel or under an infrared lamp to prevent hypothermia. While exposure to water in an inescapable situation requires physical responses, it constitutes a strong aversive stimulus particularly for mice. FST produces activation of neuroendocrine stress systems and stress-related gene expression [16]. Cold swim stress is a variant of the FST that exposes rodents briefly to a water cooler than used in FST (~18°C) [17]. Both swim protocols represent brief but severe psychophysiological stressors.

#### 2.1.3 Acute Social Defeat Stress (ASDS)

The model relies on the motivation of male rodents to dominate their territory by excluding unfamiliar intruders. In acute social defeat stress (ASDS), the intruder mouse or rat is exposed to a resident conspecific in a controlled setting and removed after defeat when the intruder displays submissive behavior. Residents are typically chosen from an aggressive strain and are single housed for several days prior to ASDS. The test subject (intruder) is introduced to the cage of the resident, and agonistic interactions are observed by the experimenter and interrupted when necessary to prevent serious injuries. As females do not exhibit territorial aggression, this paradigm is established only in male rodents. Likewise, not all strains can be used as intruders or residents due to their trait aggression levels [18]. Although a strong acute neuroendocrine response after ASDS is reported [19], a depressive-like phenotype is achieved only after repeated sessions. ASDS is typically used as a stressor in the chronic unpredictable mild stress (CUMS) protocol [20].

### 2.2 Chronic Stress (Adult)

#### 2.2.1 Chronic Unpredictable Mild Stress (CUMS)

Over a period of weeks, the animal is exposed to a variety of different minor stressors such as food and water deprivation, tilting of the cage, stroboscopic light and others. Due to the chronically elevated physiological stress response, a depressive-like phenotype emerges that can be measured in the sucrose preference test (SPT) and the FST. Furthermore, this phenotype is subject to reversal by a wide range of antidepressant drugs [21].

#### 2.2.2 Repeated Restraint Stress (RRS)

Repeated restraint stress (RRS), the application of an acute restraint procedure lasting from several days to weeks, has produced inconclusive results. While certain biological stress responses increase with the number of restraints, others return to basal levels and show no sensitization [22]. The heterogeneity of protocols, specimens studied and the lack of sampling during the protocol continue to pose significant problems in interpreting the physiological stress response [23].

#### 2.2.3 Chronic Social Defeat Stress (CSDS)

Chronic social defeat stress (CSDS) is commonly used as a paradigm to model depression-like behavior [24]. In repeated confrontational encounters with an aggressive resident, the intruder's hypothalamic-pituitary-adrenal (HPA) axis is continuously activated and results in a depressive-like phenotype characterized by anhedonia, anxiety-related behavior and social avoidance. Although there is inherent variability in social interactions in rodents, it should be noted that social stressors constitute a severe form of stress [25].

#### 2.2.4 Chronic FST

Although rarely used, in chronic FST rats are exposed to forced swimming daily for a few minutes for extended periods of time. Whereas nerve growth was observed in the limbic areas of the HPA axis, no study has demonstrated effects on the HPA axis function itself [26].

### 2.3 Early Life Stress

#### 2.3.1 Maternal Separation

Early life stress seeks to manipulate the postnatal environment of dams and littermates and thus the long-term behavioral and physiological development of the offspring. More specifically, maternal separation disrupts the infant-mother relationship by the separation of the litter from the dam, which constitutes a strong stressor to the pups. As a result, the pups lack the daily nutrients and attention provided by the dam and, later in life, are expected to develop depressive-like symptoms, including anhedonia and passive/submissive behavior, as well as increased anxiety-related behavior [27, 28]. The protocol typically is carried out in the first two weeks of the pup's life. The pups may be placed in an unfamiliar cage, or the home cage. Separation time typically ranges from 2-8 hours. Care must be taken to avoid hypothermia in the pups. Also, non-handling, the complete absence of handling, is usually not recommended as a control group since this treatment also can affect the offspring. Therefore, animal-facility rearing is the control group of choice, with weekly cage changing and minimal handling [29]. The prolonged separation leads to physiological and emotional stress in the offspring. While maternal separation is thought to reduce the time of maternal care and infant-dam interactions, a protocol of minimal bedding and limited nesting material from post-natal days 2-9 results in fragmented maternal care and reduces its overall quality [30].

## 3 HOW TO MEASURE STRESS OUTCOMES IN RODENTS?

### 3.1 The Hypothalamic-pituitary-adrenal (HPA) Axis-centered Stress Response

The HPA axis is the main neuroendocrine mechanism of the body’s adaptation to stress [31]. Upon exposure to a stressor, the activation of the sympathetic nervous system and the HPA axis, leads to the release of catecholamines and corticotropin-releasing hormone from the hypothalamus, which in turn results in the secretion of the adrenocorticotropic hormone from the pituitary gland [32]. The stress-induced activation of the HPA results in a release of corticosterone in animals, which affects neural signaling through mineralocorticoid and glucocorticoid receptors [11]. Ultimately, the multifaceted response of the stress regulatory systems seeks to restore the homeostasis of the organism [33].

### 3.2 Behavioral Testing

Due to the multifactorial coordination of the stress system, it is challenging to obtain a single readout of responses to stress by testing animal behavior. Overall, a variety of tests need to be conducted to get a broader picture of the animal’s response. While responses of the endocrine system may be orchestrated by defined molecular cascades, the behavioral response is likely to be adapted to a certain type of stressor and thus may vary between different stress protocols. Protocols for chronic stress aim at altering the functioning of the animals' stress response persistently.

#### 3.2.1 Elevated Plus Maze (EPM)

The elevated plus maze (EPM) is the most widely used test for anxiety-like behavior and for the screening of anxiolytic compounds [34]. Rodents display innate aversion to elevated, open and brightly-lit spaces and preference for enclosed, dark spaces, respectively. The animals are introduced to the EPM apparatus, where their unconditioned response to these stimuli is measured. The EPM consists of four arms arranged in a cross, where two opposite arms are enclosed by walls and the other two are open and brightly illuminated. Time and number of entries to the open arms are used to measure anxiety-related responses. Treatment with anxiolytics has been reported to increase time and entries in the open arms [35, 36].

#### 3.2.2 Open Field Test (OFT)

In the open field test (OFT), the animal is introduced to a large area with walls and left to explore for several minutes. Typically, the OFT is divided into an outer and a central area. Rodent innate avoidance of open spaces and their tendency to move alongside the wall (thigmotaxis) presents them with a choice to remain close to the walls or explore the central area of the open field. In addition to locomotion, a number of parameters such as rearing, defecation, number of entries to and time in the inner zone are recorded. OFT is commonly used to assess (hyper- or hypo-) locomotion in pharmacological studies, as locomotion itself is also considered a measure of anxiety-like behavior. Additionally, entries to and time in the inner zone are used to measure anxiety-like behavior [37]. Large variability of experimental conditions, *e.g*., light intensity, open field size and shape, as well as recording methods, are reported [18]. Pharmacological studies have shown varying results concerning changes in locomotion and time spent in the central area after different anxiolytic treatments [38].

#### 3.2.3 Forced Swim Test (FST)

Tests for depressive-like behavior, such as the FST, rely on the observation that animals in an inescapable situation display immobility behavior. The duration and onset of these behaviors can be reduced and delayed, respectively, by the application of antidepressant compounds. Initially developed for rate use, the original test consists of two trial sessions of several minutes, where the animal is introduced to a tank with water from which it cannot escape. The first session serves to habituate the animal to the test. In a second session, the animal behavior is recorded. In mice, only one session is used. Following initial active movements, the animal displays episodes of immobility, where movement, except for those necessary to keep its head above water, ceases [39, 40]. Although the usefulness of screening antidepressant drugs and compounds is not debatable, the interpretation of the immobility parameter as depressive-like behavior has come into question, with an increasing number of studies favoring immobility as a coping strategy [14].

#### 3.2.4 Tail Suspension Test (TST)

During the tail suspension test (TST), mice are suspended from their tails, and immobility time and latency are recorded, where immobility means the total lack of movement. The test is unique to mice and the duration is typically 6 min. Similar in theory to the FST, the TST exposes the animals to an inescapable situation, and immobility time and latency to immobility can be reduced and delayed, respectively, by antidepressant treatment [40].

#### 3.2.5 Social Interaction Test (SIT)

Chronic stress may lead to reduced sociability, in particular, as an outcome of the CSDS protocol. Social withdrawal specifically is a symptom of depressive disorders, therefore, corresponding animal models may provide valuable tools to establish reduced sociability in rodents. Measurements of sociability are based on the preference of rodents to spend time with a conspecific rather than being alone, or on the preference of a social *vs.* a non-social stimulus. In the social interaction test (SIT), pairs of animals are introduced to a novel environment, typically a new cage, and their exploratory and social behaviors are recorded. Given the difficulties in quantifying the social behaviors in this setup, a number of tests have been developed which place the conspecific stimulus mouse in a confined space while letting the test subject explore. Thus, avoidance and social interactions can be measured [41].

#### 3.2.6 Sucrose Preference Test (SPT)

Anhedonia, the inability to experience pleasure or reward from positive events, is considered a key symptom of depression and has been reported to occur after CUMS [42]. Animals failing to show a preference for sucrose (or saccharin) is indicative of their anhedonic state. Typically, after habituation to two bottles with tap water, the animals are presented with two bottles, one containing tap water and one bottle containing a defined sucrose solution, for one to several hours. Sucrose preference is expressed as (sucrose intake)/(total intake). SPT takes place in the home cage, after a certain time of food and/or water deprivation [43]. Care must be taken to match body weight and sucrose intake between treatment and control groups. Since single housing is required in this paradigm, female mice and rats are less adequate as test subjects as this housing condition poses an additional stressor to them [44]. Based on SPT outcomes, animals can be separated into SPT-susceptible and SPT-resilient.

### 3.3 Molecular Readouts

In response to stressful stimuli, cortisol in humans and corticosterone in rodents are secreted from the adrenal glands, and *via* its binding to the mineralocorticoid and glucocorticoid receptors in the brain and the periphery, mediates the termination of the HPA axis activation and maintenance of the homeostasis [32]. Glucocorticoids exert their effects at the hypothalamus and pituitary *via* a closed-feedback loop mediating the stress response [45]. Corticosterone, the main glucocorticoid of rodents, has long been considered a stress indicator [46-48] which is relatively easy to measure with kits and/or radioimmune assays. Corticosterone concentrations depend on the stressor type and intensity as well as on the time interval between stress exposure and sampling [49]. Commonly, corticosterone is estimated in serum or plasma. Such concentrations represent the total glucocorticoids found in plasma or serum, *i.e*., both the levels of free glucocorticoids and the globulin-bound glucocorticoids. It has been suggested that only the free glucocorticoids are actually active, thus the concentrations measured do not represent their biologically active forms [31, 50]. Corticosterone metabolites can also be measured in feces and urine, with these procedures being less invasive. However, such concentrations usually refer to corticosterone metabolites, since feces or urine contain little to no corticosterone [47, 51].

## 4 METABOLOMICS AND PSYCHOPATHOLOGIES

Holistic approaches towards disentangling complex biosignatures in psychiatric phenotypes have emerged using -omics methodologies to analyze rodent models and human cohorts [52-55]. Concerning stress-related phenotypes in rodent models, the effects of cold swim stress were studied by protein-, phosphoprotein- and RNA-based -omics approaches in mouse hippocampus, indicating a stress-induced, time-dependent network of orchestrated alterations in protein translation machinery [56]. Furthermore, distinct multiomics signatures upon stress exposure were observed in mouse dorsal *vs.* ventral hippocampus using three different acute stress paradigms [57, 58]. Quantitative proteomics has pinpointed that CUMS, in the absence of the cytoskeletal Tau protein, is mediated by synaptic mitochondria in the mouse prefrontal cortex [59], whereas CSDS was shown to impair the function of the glucose metabolism enzyme hexokinase 3 in brain mitochondria-associated membranes [60].

The metabolome constitutes the first line of defense in an organism and is particularly sensitive to environmental changes and external stimuli [61]. Metabolomics, a recent addition to the -omics family, is tailored towards analyzing small molecules by two main approaches: targeted metabolomics measuring *a priori* selected metabolites [62] and untargeted metabolomics investigating metabolomic profiles in a hypothesis-free manner [63]. Methodologically, the main technologies used in metabolomics are mass spectrometry (MS), either coupled with liquid or gas chromatography (liquid chromatography-tandem mass spectrometry (LC-MS/MS) and gas chromatography-mass spectrometry (GC-MS), respectively) and nuclear magnetic resonance (NMR). For comprehensive reviews on metabolomics methodologies and approaches, we direct the readers to [64-66]. Metabolomics studies have been widely implemented in preclinical and clinical neuropsychiatric research [67, 68], including, among others, anxiety disorders [69], post-traumatic stress disorder [70] and depression [71]. We have previously used metabolomics approaches to study altered molecular profiles in mouse models of schizophrenia-like symptoms [72] and high anxiety-related behavior [73-75]. We have also looked for metabolome alterations upon treatment with compounds with antidepressant [76-79] and anxiolytic [80] properties in mice. In patients suffering from psychiatric disorders, metabolomics has been predominantly used to delineate metabolome signatures in peripheral material, such as serum [11] and plasma [81].

## 5 AIM OF THE REVIEW

Here, we review metabolomics studies in rodents (mouse and rat) that have been exposed to acute, chronic and early life stress paradigms. We have included studies (summarized in Table **1**) which: i. focus on brain tissue but also on peripheral material (serum and plasma) due to its diagnostic and translational potential ii. use the terms metabolomics or metabonomics to describe the methodological approach employed and iii. implement both targeted and untargeted MS- and NMR-based metabolomics platforms. We do not expand on aggression-related, social hierarchy paradigms in rodents such as dominance/subordination. For these paradigms, we direct the interested reader to published work on hierarchical status-induced metabolomic signatures [82]. We have not included studies addressing environmental/dietary/toxi-cological stressors. We have not considered studies investigating stress responses along with confounding pathologies or other interventions besides stress (*e.g*., aquapuncture, diet, learning and memory paradigms, herbs, stress hormone supplementation), pharmacological studies, studies measuring selected metabolites with high performance liquid chromatography (HPLC), urine or saliva studies and studies using post-traumatic stress disorder rodent models.

## 6 METABOLOMICS STUDIES IN RODENT MODELS OF ACUTE STRESS

### 6.1 Acute Restraint Stress (ARS)

#### 6.1.1 Brain Tissue

Brain metabolomics studies of ARS effects are scarce. In the only study that, to the best of our knowledge, could be found young adult (9 weeks old) male C57BL/6J mice were restrained for 30 min, and selected amino acids (glutamate, glutamine and GABA) were quantified by LC-MS/MS, 30 min after the end of the restraint [83]. Glutamate and glutamine, but not GABA, levels were significantly increased in the prefrontal cortex of restrained mice compared to the control group. No altered levels of the aforementioned metabolites were observed in the hippocampus between the two groups.

#### 6.1.2 Peripheral Material

Recently, mitochondria have gained increased attention in the context of stress [84]. To investigate the role of diverse mitochondrial pathways in the acute stress response, ARS effects were studied in 10-13 months old male mice, mutated for either nuclear or mitochondrial DNA encoded mitochondrial genes and wild type mice of C57bl/6eij background [85]. Each mouse model included one of the following mutations or deletions: deletions in nuclear DNA-encoded genes of either adenine nucleotide translocator 1 or nicotinamide nucleotide transhydrogenase, as well as mutations in mitochondrial DNA-encoded genes of either NADH dehydrogenase 6 or cytochrome c oxidase subunit I. To assess HPA axis reactivity, plasma corticosterone and adrenocorticotropic hormone levels were measured. Then, LC-MS analysis was conducted to determine catecholamine plasma levels, including norepinephrine, epinephrine, dopamine and serotonin, after 60 min of restraint in stressed *vs.* their basal levels in the pre-stress state (control state) in the aforementioned mouse models. Wild type restrained mice showed significantly increased circulating levels of norepinephrine, epinephrine and dopamine compared to wild type controls. In the plasma of nicotinamide nucleotide transhydrogenase-deficient mice after restraint, only norepinephrine and dopamine levels were increased compared to the basal pre-stress state. NADH dehydrogenase 6- as well as adenine nucleotide translocator 1-deficient mice showed elevated dopamine levels upon restraint, whereas cytochrome c oxidase subunit I-deficient mice had increased levels of all 4 catecholamines investigated (norepinephrine, epinephrine, dopamine and serotonin) after restraint.

Teague *et al.* restrained young, adult male Sprague-Dawley rats for 6h and investigated the plasma metabolic status 0, 1, 3 and 6h after restraint initiation by ^1^H NMR [86]. The plasma metabolic profile at the 0h time point was considered to be the control state. Εlevated levels of glucose and acetone were measured in all timepoints (1 h, 3h and 6 h) compared to control 0 h. β-hydroxybutyrate (ketone bodies) and glutamine levels were increased only after 3h of restraint compared to 0h controls. The levels of acetate, lactate, lipoproteins and those of the amino acids alanine, isoleucine, leucine, and valine were reduced after restraint at all time points studied compared to the control 0h time point. Overall, these results indicate that ARS impacts on glucose metabolism and amino acid catabolism. Ketone bodies participate in ATP and neurotransmitter production in the brain [87, 88] and, recently, it was found that ketone body utilization in the brain is activated upon acute stress exposure in mouse prefrontal cortex [83]. Teague *et al.* additionally compared plasma metabolome alterations in response to acute and repeated restraint stress exposure (‘Repeated restraint stress (RRS)’, peripheral material).

### 6.2 Forced Swim Stress (FST)

#### 6.2.1 Brain Tissue

It has been shown that FST affects HPA axis reactivity in a sex-dependent manner [16]. To investigate whether steroid levels in females and males are differentially affected upon acute stress in a brain region-specific manner, Sze *et al.* exposed 21-week-old Sprague-Dawley rats of both sexes to a 2 min FST protocol [89]. Rats were sacrificed 30 min after FST initiation and the levels of 8 steroids (corticosterone, deoxycorticosterone, dihydrodeoxycorticosterone, pregnenolone, progesterone, dihydroprogesterone, allopregnanolone and testosterone) were measured by LC-MS/MS in the frontal cortex, hypothalamus, hippocampus, amygdala, brainstem and plasma. In all brain regions of both male and female stressed rats, corticosterone, deoxycorticosterone and progesterone levels were higher compared to unstressed, sex-matched controls. Additionally, stressed females showed increased dihydroprogesterone and pregnenolone levels in all brain regions compared to control females. However, dihydroprogesterone levels were elevated only in the frontal cortex and brainstem, while pregnenolone levels were increased in all regions studied except for the hypothalamus of stressed compared to unstressed control males. Dihydrodeoxycorticosterone levels were also significantly increased in all investigated brain regions besides the amygdala in stressed females, while stressed males showed increased levels of dihydrodeoxycorticosterone only in the frontal cortex compared to the respective sex-matched controls. Allopregnanolone levels were higher in the male frontal cortex only and in the female frontal cortex, amygdala and brainstem *vs.* corresponding unstressed controls.

Although the cerebellum has been traditionally involved in motor functions, recent data have implicated this brain region in stress responses [90]. Upon FST acute stress for 6 min, we have found cerebellar metabolome alterations in male 7-week-old CD1 mice compared to unstressed counterparts 2h after FST initiation [91]. NMR-based metabolomics analysis identified 47 known metabolites, of which 19 had altered levels between stressed and control groups. Pathway enrichment analysis highlighted significant enrichment in aspartate, purine, glutamate, and amino sugar metabolism, as well as urea cycle and ammonia recycling. We also found significant correlations between FST parameters (swimming and floating time) and metabolite levels of N-acetylaspartic acid, UMP, threonine and tyrosine in stressed mice.

Bassett *et al.* in their study investigated metabolome level differences in the brain and plasma of two rat strains, the stress-vulnerable Wistar Kyoto strain and the normative anxiety Sprague-Dawley strain, upon FST exposure (see also ‘Forced swim test (FST)’, peripheral material) [92]. Behavioral phenotyping of anxiety-related and depression-like behavior included OFT and FST, with FST also being used as the stressor. Wistar Kyoto rats showed increased anxiety-related and depression-like behavior compared to Sprague-Dawley rats in OFT and FST, respectively. However, among the 38 annotated polar metabolites, no metabolite level differences were found using hydrophilic interaction chromatography (HILIC)-MS analysis as whole brain polar metabolite levels did not reach significance between stressed and control groups for either Wistar Kyoto or Sprague-Dawley rats.

#### 6.2.2 Peripheral Material

Shi *et al.* investigated altered plasma metabolomic signatures in three different paradigms of stress-induced depression-like behavior [93]. Male Sprague-Dawley rats were subjected to either acute FST for 5 min, chronic FST for 14 days (‘Chronic FST’) or CUMS for 4 weeks (‘Chronic unpredictable mild stress (CUMS)’) and metabolite level alterations upon stress exposure compared to unstressed mice were investigated within each protocol as well as across protocols using NMR-based metabonomics. Rats belonging to the acute FST group were forced to swim for 5 min, and 24h after the end of the session animals were sacrificed. Trimethylamine levels were significantly decreased in the plasma of the acute FST *vs.* the control group.

Bassett *et al.* as well as Sze *et al.* investigated both brain and plasma metabolome differences in rats upon FST exposure with HILIC-MS [92] and HPLC-MS [89] metabolomics, respectively. In Bassett *et al.*, although no significant differences were found in the whole brain metabolome upon stress exposure, decreased levels of glutamic acid, 3-methoxytyrosine, cytosine and GABA were reported in the plasma of stressed Wistar Kyoto rats compared to controls [92]. In Sze *et al.*, plasma steroid levels analysis upon FST exposure showed that a 2 min FST session followed by 30 min recovery time increased corticosterone, deoxycorticosterone and progesterone levels in both sexes [89].

### 6.3 Acute Social Defeat Stress (ASDS)

#### 6.3.1 Brain Tissue

There is a growing body of interest in molecular pathways that shape stress resilience. In their study, Dulka *et al.* used ASDS to investigate brain metabolomic alterations in adult C57BL/6 male mice [94], which were exposed three times for 2 min each to the home cage of three different resident aggressor CD1 mice. 24h after the last encounter, defeated mice were categorized into stress-susceptible and stress-resilient based on their performance in the SIT, with stress-susceptible mice having a significantly reduced social interaction ratio compared to stress-resilient and non-defeated groups. To investigate ASDS-induced neurochemical activity alterations, mice were again subjected to an additional social defeat stress exposure one week following the SIT and immediately after, the basolateral/central amygdala, dorsal hippocampus, nucleus accumbens and ventromedial prefrontal cortex were obtained for untargeted metabolomics by ultra-high performance liquid chromatography-high-resolution mass spectrometry (UPLC-HRMS). In the dorsal hippocampus, stress-resilient mice had a lower relative abundance of GABA compared to stress-susceptible mice. Moreover, cysteine levels in the nucleus accumbens of stress-resilient mice were increased compared to stress-susceptible mice. In the ventromedial prefrontal cortex, stress-resilient mice showed elevated levels of inosinic acid and AMP compared to stress-susceptible mice. Therefore, distinct metabolic pathways synergize in each brain region to confer resilience or vulnerability to stress.

## 7 METABOLOMICS STUDIES IN RODENT MODELS OF CHRONIC STRESS

### 7.1 Chronic Unpredictable Mild Stress (CUMS)

#### 7.1.1 Brain Tissue

The identified metabolomics studies using CUMS focused on adult male rats. An untargeted GC-MS metabolomics approach in multiple organs and serum of 8-week-old Sprague-Dawley rats which underwent CUMS for 4 weeks [95] revealed 4 metabolites with altered levels in whole brain extracts in CUMS compared to the control group, namely cholesterol, D-lactic acid, carbamic acid and stearic acid. Analyzing the same cohort with untargeted UPLC-MS metabolomics, a previous study of the same group investigated metabolite level alterations in the hippocampus of 8-week-old Sprague-Dawley rats exposed to a 4-week CUMS protocol [96]. The CUMS group showed decreased sucrose consumption in the SPT and increased immobility time in the FST, overall indicating an increased depression-like behavior compared to the control group. 35 metabolites with altered levels between CUMS and control groups were identified. Integrated metabolomics and lipidomics analysis highlighted CUMS-affected pathways related to glycerophospholipids, glycine, serine and threonine metabolism, phenylalanine metabolism as well as alanine, aspartate and glutamate metabolism.

Li *et al.* as well as Zhang *et al.* used CUMS to investigate brain metabolome alterations in a rat CUMS-induced model of depression [97, 98]. In these two studies, adult Sprague-Dawley rats were exposed to a 3-week CUMS protocol. Depression-like behavior was assessed prior to stress exposure and weekly during the CUMS protocol *via* the SPT. In both studies, rats were divided into stress-susceptible and stress-resilient based on the sucrose consumption in the final SPT and stress-susceptible *vs*. unstressed control rats were compared by MS-based metabolomics. Post-CUMS behavioral phenotyping assessed by OFT, EPM and FST indicated increased anxiety- and depression-like behavior in the stress-susceptible group compared to the control group [97]. In the amygdala, LC-MS/MS metabolomics analysis revealed altered levels of 42 metabolites between stress-susceptible and control groups, which were mainly related to amino acid metabolism. Hippocampal metabolome alterations upon 3 weeks of CUMS exposure investigated by GC-MS followed by multivariate analysis showed altered levels of 30 metabolites, among the 363 metabolite compounds that were identified, between stress-susceptible and control rats, with most of them being associated with amino acid and lipid metabolism [98].

Hippocampal metabolome alterations in response to different stress protocols, including CUMS, RRS (see 'Repeated restraint stress (RRS), brain tissue'), learned helplessness and social defeat, were investigated by Liu *et al.* [99]. Effects of a 3-week CUMS exposure in adult male Sprague-Dawley rats were assessed by GC-MS metabolomics. Once per week during the CUMS protocol, as well as before and 24h after CUMS exposure, the SPT was performed. Anxiety- and depression-like behavior was evaluated by OFT, EPM and FST. Similarly to other studies, CUMS rats were divided into stress-susceptible and stress-resilient according to the SPT, and comparisons were made only between stress-susceptible and unstressed control rats. Stress-susceptible rats showed increased depression-like and anxiety-like behavior after 3 weeks of CUMS exposure. Untargeted GC-MS metabolomics in the hippocampus indicated 30 metabolites with altered levels between stress-susceptible and control rats. Affected metabolites were associated with lipid and glutamate metabolism, molecular transport, inflammatory responses and small molecule biochemistry, as indicated by pathway and network analysis.

Linhu *et al.* [100] used the stable isotope-resolved metabolomics (SIRM) approach implementing stable isotopes of glucose and investigated hippocampal alterations in energy metabolism pathways, upon CUMS exposure in adult male Sprague-Dawley rats with the same experimental procedure described previously in the periphery (see ‘Chronic unpredictable mild stress (CUMS), peripheral material’). Of the 46 identified metabolites, 22 showed altered levels between the CUMS and control groups. Results pinpoint tricarboxylic acid cycle inhibition and gluconeogenesis activation in the hippocampus of the CUMS compared to the control group. In another study, a 4-week CUMS protocol was applied to 8-week-old Sprague-Dawley male rats and metabolomic changes in the cerebral cortex, hippocampus, thalamus, and the remaining brain regions were analyzed by GC-MS [101]. CUMS rats are characterized by increased depression-like behavior compared to the control group in the SPT. Μultivariate analysis revealed 24 annotated metabolites accountable for class discrimination in one or more brain regions studied and were mostly related to amino acid metabolism.

Metabolome alterations in the prefrontal cortex of male adult Sprague-Dawley rats upon 4 weeks of CUMS exposure were investigated by Duan *et al.* [102] by LC-MS/MS metabolomics. CUMS rats showed increased depression-like behavior in the SPT and FST compared to the control group. Metabolomics data analysis indicated 5 metabolites with altered levels between the CUMS and control group, namely 1-methylnicotinamide, 3-methylhistidine, acetylcholine, glycerophospho-N-palmitoyl ethanolamine and α-D-mannose 1-phosphate. These metabolites are predominantly involved in nicotinate and nicotinamide metabolism.

#### 7.1.2 Peripheral Material

Besides various organs, including the brain, Geng *et al.* [95] (see also 'Chronic unpredictable mild stress (CUMS), brain tissue’) investigated rat serum upon CUMS. Serum untargeted GC-MS metabolomics identified 10 metabolites with altered levels between CUMS and control groups. These metabolites are mainly implicated in alanine, aspartate and glutamate metabolism, phenylalanine, tyrosine and tryptophan biosynthesis, D-glutamine and D-glutamate metabolism, arginine and proline metabolism and linoleic acid metabolism.

To focus on serum metabolites that are associated with energy metabolism, LC-MS/MS based, untargeted metabolomics (HILIC and T3 column LC-MS) and SIRM using glucose stable isotopes were used in a rat model of CUMS [103]. 8-week-old male Sprague-Dawley rats were subjected to a 28-day CUMS protocol and weekly behavioral screening pinpointed an increased depression-like behavior in the CUMS group. LC-MS/MS-based metabolomics analysis identified 50 metabolites with altered levels in the CUMS compared to the control group that were mainly related to energy metabolism. Pathway analysis showed that valine, leucine and isoleucine biosynthesis, arachidonic acid metabolism, pyruvic acid metabolism, alanine, aspartate and glutamate metabolism, glycerophospholipid metabolism, cysteine and methionine metabolism, glycolysis or gluconeogenesis, tricarboxylic acid cycle, and purine metabolism were among the most affected pathways due to CUMS. SIRM analysis revealed that among the 78 labeled metabolites, 28 were significantly different between the two groups. Intriguingly, it was hypothesized that the increased pyruvic acid levels in the CUMS group *vs.* controls, could be a result of the slow downstream metabolism of pyruvic acid, and the authors suggest that CUMS induced a tricarboxylic acid cycle block. Moreover, the authors support that CUMS also activates the gluconeogenesis pathway, resulting in excessively elevated pyruvic acid levels that participate in the pyrimidine biosynthesis pathway, phospholipid synthesis pathway, and amino acid metabolism.

In plasma, adult Sprague-Dawley rats were subjected to a CUMS protocol for 4 weeks [104]. Plasma UPLC-MS metabolomics analysis indicated that in the CUMS group, 30 metabolites showed altered levels compared to the control group, including amino acids, fatty acids, carnitines and phospholipids, suggesting that CUMS impacts multiple metabolic pathways/functions related to amino acid metabolism, ATP supply, inflammation and cell membrane integrity.

To identify stress type-specific plasma metabolic alterations in acute and chronic stress rat models, adult male Sprague-Dawley rats were exposed either acutely or chronically to FST ‘Forced swim stress (FST)’ and ‘Chronic FST’) and to CUMS for 4 weeks [93]. Rats were tested weekly in the SPT as well as the OFT to assess depression-like and anxiety-like behavior and locomotion. CUMS rats are characterized by increased depression and anxiety-like behavior compared to the control group. Plasma metabolome was analyzed by NMR-based metabolomics and 12 metabolites were selected as potential stress markers based on PLS-DA analysis and parametric statistics. Trimethylamine, aspartic acid, glutamate, acetoacetic acid, N-acetyl glycoprotein, alanine, lactate, leucine/isoleucine and lipids levels were elevated, while proline, β-hydroxybutyric acid and valine levels were reduced in the CUMS compared to the control group. Overall, the authors support that CUMS affects glutamate and energy metabolism as well as inflammatory responses. Multivariate analysis showed that specific metabolite groups could be used to differentiate between each stress model.

### 7.2 Repeated Restraint Stress (RRS)

#### 7.2.1 Brain Tissue

Li *et al.* [105] subjected 25 adult male Sprague-Dawley rats to RRS, 8h daily for 21 consecutive days, and compared them to 25 controls. Following RRS, body weight as well as rat behavior in the OFT were assessed. RRS decreased body weight and increased anxiety-related behavior in the OFT. The metabolomes of stressed and control mice were compared with GC-MS metabolomics, revealing 11 metabolites with altered levels, of which 6 were found in higher (cholesterol, glycine, methyl phosphate, 2-butyne-1,4-diol, dCTP and 2-amino-3-methyl-1-butanol) and 5 in lower (GABA, threonine, inosine glucose-1-phosphate and N(epsilon)-trimethyllysine) levels in the amygdala of RRS rats compared to controls. Pathway analysis indicated that the aforementioned metabolites are involved in amino acid and energy metabolism, as well as nucleotide metabolism and translation.

Liu *et al.* stressed Sprague-Dawley rats for 6h daily for 21 days, while control rats were left undisturbed. After RRS, animals were subjected to a behavioral test battery (SPT, OFT, EPM, FST). Animal body weight was also assessed and was found significantly lower in RRS animals compared to controls, after the RRS protocol. FST immobility time was significantly higher in RRS rats compared to controls, highlighting an increased depression-like behavior. Following RRS, 8 rats were classified as stress-susceptible and 12 as stress-resilient based on the SPT and only the stress-susceptible rats were used for metabolomics comparisons with control rats, during which prefrontal cortex tissues were analyzed by GC-MS. 36 metabolites with significant level differences between RRS and control rats were identified in the prefrontal cortex, including among others myo-inositol, glutamine, trehalose-6-phosphate and L-allothreonine. Pathway analysis showed that the above 36 metabolites were predominantly involved in anandamide degradation, tRNA charging, uracil degradation, asparagine biosynthesis and stearate biosynthesis. Network function analysis pinpointed that the most significantly altered networks were lipid metabolism and small molecule biochemistry [106].

In a later study of the same group [99], the authors applied a series of environmental stressors to 140 male Sprague-Dawley rats, including CUMS (see ‘Chronic unpredictable stress (CUMS), brain tissue’), learned helplessness, RRS and social defeat. Specifically for RRS, mice were restrained for 6h daily for 21 days, while being deprived of food and water. Behavior was characterized by SPT, FST, OFT and EPM. Hippocampi samples were collected and analyzed by GC-MS metabolomics, revealing nine metabolites with altered levels between RRS and control rats. The perturbed pathways were involved in sphingosine and sphingosine-1-phosphate metabolism and four metabolites, namely lactic acid, N-acetyl-L-aspartic acid, phosphorylethanolamine and phosphate, were found to be involved in cellular growth and proliferation, organismal development and nervous system development and function.

#### 7.2.2 Peripheral Material

Chen *et al.* [107] examined the plasma metabolomic profiles of RRS in rats. Rats were subjected to RRS for ten days, 1h daily at unpredictable times. Behavior was assessed by the EPM one day before the beginning and also on the last day of RRS. At the beginning of the experiment, time spent in the open arms of the EPM was similar for both the RRS and control rats, while on day 10 of the experiment, the stressed rats spent significantly less time in the EPM open arms compared to the control rats. RRS also resulted in weight loss. The plasma metabolic profiles of the RRS and control groups were assessed by ultra-high-performance liquid chromatography-triple quadrupole-time-of-flight mass spectrometry (UPLC-QTOF-MS). Seventeen plasma metabolites with altered levels were identified, of which L-methionine, L-carnosine and glyceric acid were found in lower levels, while the rest were found in higher levels in RRS compared to control rats. The aforementioned 17 metabolites were involved in histidine metabolism, neurotransmitter synthesis and bile acid metabolism.

In a similar approach, Teague *et al.* [86] used ^1^H NMR plasma metabolomics to assess the effects of RRS in 5 young adult male Sprague-Dawley rats. The animals were exposed to RRS for 6h per day for 35 consecutive days. The stressor consisted of a restraint wire mesh, shaking and restraint plus shaking and started at 10:00 while stressors changed every hour during the 6h stress session for minimizing habituation and adaptation. Stress induced perturbations at both the low molecular weight plasma metabolites, as well as at the lipidomic profiles compared to pre-stressed animals. Specifically, the ^1^H NMR spectra of rat plasma after RRS, which were measured on days 1, 9, 21, 35 and 44 of the experiment, showed an increase in the levels of lactate, alanine and choline and a decrease in the levels of very-low-density lipoproteins/low-density lipoproteins upon RRS. Furthermore, as chronic stress progressed, there a decrease in plasma acetate levels was reported. Also, a marked increase in choline and glycerol levels and a decrease in plasma lipidomic profiles was observed.

### 7.3 Chronic Social Defeat Stress (CSDS)

#### 7.3.1 Brain Tissue

Brain CSDS metabolomics studies have focused on brain regions primarily involved in emotional processing, such as the prefrontal cortex, hippocampus and amygdala nuclei. In a CSDS mouse model, adult (7-8 weeks) male C57BL/6J mice were subjected to a 10-day CSDS protocol [108]. Defeated mice were separated into stress-susceptible and stress-resilient according to the social interaction ratio evaluated 24h after the last CSDS exposure. Metabolomics analysis was conducted in the medial prefrontal cortex, nucleus accumbens, ventral hippocampus and the plasma (see ‘Chronic social defeat stress (CSDS), peripheral material’), using GC/MS and reverse phase LC/MS and HILIC-LC-MS/MS). Unsupervised analysis showed altered levels of 39, 7 and 19 metabolites in the ventral hippocampus, medial prefrontal cortex and nucleus accumbens, respectively, between the CSDS groups and the control group. Pathway analysis revealed perturbations in fatty acid beta oxidation, antioxidant defense and purine metabolism. Notably, distinct metabolite level signatures were found in each group (stress-susceptible and stress-resilient). Also, untargeted metabolomics identified 34 and 11 metabolites with significantly altered levels in the ventral hippocampus and the nucleus accumbens, respectively, suggesting a brain-region specific metabolic effect of CSDS in stress-susceptible and stress-resilient groups compared to controls. In the ventral hippocampus, the identified altered metabolites were found mainly in stress-susceptible animals *vs.* controls, while in the nucleus accumbens, metabolite level changes occurred in stress-resilient *vs.* controls. Therefore, the authors suggest that the metabolic effect of chronic stress in the nucleus accumbens is related with stress-resilience, whereas in the ventral hippocampus is associated with stress-susceptibility.

It has been proposed that fluctuations in the blood-brain barrier integrity are associated with depression and stress-resilience. Perturbed expression of endothelial tight junction proteins is observed in the nucleus accumbens of patients suffering from depression and is modulated by antidepressant treatment [109]. To investigate metabolome changes underlying blood-brain barrier breaks, Zhang *et al.* [110] subjected adult, male C57BL/6J mice to a 10-day CSDS protocol and 24h after the last defeat, mice were separated into stress-resilient and stress-susceptible according to the SIT. Stress-susceptible mice showed increased depression-like behavior in the TST compared to stress-resilient and control mice. 24h after the SIT, mice were sacrificed and the nucleus accumbens, prefrontal cortex and hippocampus were analyzed with GC-MS and LC-MS/MS metabolomics, where 255 metabolites were overall identified. Significantly reduced cAMP levels were observed in the nucleus accumbens in the stress-susceptible group compared to controls. A positive correlation was highlighted between nucleus accumbens cAMP levels in all three groups and the social interaction ratio in the SIT. Νo metabolite level alterations were identified between the stress-resilient, stress-susceptible and control groups in the prefrontal cortex and hippocampus.

Additional studies have focused on the prefrontal cortex and the hippocampus of CSDS mice [111, 112]. Wang *et al.* applied a 10-day CSDS protocol to 2-month-old C57BL/6 male mice [111]. Six hours after the last defeat, mice were subjected to a behavioral test battery to assess social aversion, depression-like and anxiety-like behavior by SIT, SPT and OFT, respectively, and mice were grouped according to their social interaction scores into stress-resilient and stress-susceptible. Stress-susceptible mice showed increased depression- and anxiety-like behavior in the SPT and OFT. Wang *et al.* then investigated 25 metabolites related to tryptophan metabolism, GABAergic and catecholaminergic pathways after CSDS exposure in the prefrontal cortex by LC-MS/MS targeted metabolomics. Both stress-susceptible and stress-resilient mice exhibited decreased glutamate levels compared to controls. L-DOPA and vanillylmandelic acid levels were elevated in the stress-susceptible group *vs.* stress-resilient and control groups. Also, it was reported that L-DOPA levels negatively correlate with the number of entries and the distance traveled in the inner zone and the number of rearings in the OFT [112]. Moreover, there was a negative correlation between glutamate levels and sucrose consumption in the SPT. In addition, vanillylmandelic acid levels negatively correlated with the social interaction ratio as well as with the percentage of total time spent and the distance traveled in the inner zone and the number of rearings in the OFT [112].

In a follow-up study of Wang *et al.*, Xu *et al.* [112] investigated metabolome alterations in the hippocampus upon CSDS and compared their results in the hippocampus with those in the prefrontal cortex obtained by LC-MS/MS metabolomics [111] 6-8 week-old C57BL/6 male mice were exposed to the CSDS paradigm for 10 days. One day after the last defeat stress exposure, mouse sociability was evaluated by the SIT. Stress-susceptible mice showed increased depression- and anxiety-like behavior assessed by SPT and OFT, respectively, compared to controls. Hippocampal tissue was obtained for LC-MS/MS metabolomics analysis. Metabolomics results indicated that CSDS affects pathways related to tryptophan and dopamine metabolism. In particular, stress-susceptible mice had decreased 5-hydroxyindoleacetic acid and kynurenic acid as well as increased dopamine levels compared to the control group. Moreover, serotonin levels were significantly lower in the stress-resilient group compared to both stress-susceptible and control groups. Notably, different metabolites with altered levels were identified between the hippocampus and prefrontal cortex upon CSDS. Only metabolites associated with dopamine metabolism, dopamine and L-DOPA were found to be affected in both brain regions. In the hippocampus of CSDS mice (both resilient and susceptible), kynurenic acid levels positively correlated with the number of entries in the center zone and rearings in the OFT.

In a rat study [113], hippocampal metabolome alterations were observed in Sprague-Dawley males after a 21-day CSDS paradigm. Behavior after CSDS was assessed by OFT, EPM and FST, while the SPT was performed weekly throughout the CSDS protocol. CSDS rats were divided into stress-susceptible and stress-resilient based on SPT and only the stress-susceptible group was compared to unstressed controls. The stress-susceptible group was characterized by decreased sucrose preference in the SPT and increased immobility time in the FST compared to the control group, overall indicating increased depression-like behavior. No differences were observed in anxiety-related behavior between the two groups. Among the 413 metabolites identified by GC-MS metabolomics analysis, 25 metabolites were found to have significantly altered levels between the stress-susceptible and control groups. Affected hippocampal metabolites are implicated in amino acid, carbohydrate and lipid metabolism.

To investigate metabolic pathways related to depression-like behavior upon CSDS, Fan *et al.* subjected male Sprague-Dawley rats to a 3-week CSDS protocol [114]. Basal pre-stress behavior was assessed by the SPT to ensure that there would be no differences between the control and the CSDS group. 24h after CSDS, anxiety- and depression-like behavior was evaluated by SPT, OFT, EPM and FST and samples were collected 24h after behavioral screening. CSDS induced increased depression-like behavior, as indicated by the decreased sucrose preference in SPT and the increased immobility time in the FST of the CSDS group (both susceptible and resilient) compared to the control group. No differences in the anxiety-related behavior between the CSDS group (both susceptible and resilient) and controls were observed. By using LC-MS untargeted metabolomics analysis, 430 metabolites were identified in the rat amygdala, of which 37 metabolites had altered levels between CSDS (susceptible and resilient) and the control group. Enrichment analysis revealed that these metabolites were involved in D-glutamine and D-glutamate metabolism, alanine, aspartate and glutamate metabolism, arginine biosynthesis, nitrogen metabolism, biosynthesis of unsaturated fatty acids, purine metabolism and glutathione metabolism.

#### 7.3.2 Peripheral Material

Besides the brain (see ‘Chronic social defeat stress (CSDS), brain tissue’), Hamilton *et al.* [108] identified 61 metabolites with altered levels in the serum between stress-susceptible, stress-resilient and control unstressed mice, upon 10-days of CSDS in adult male C57BL/6J mice. Pathway analysis showed that affected serum pathways included nucleotide metabolism (purine/pyrimidine), energy production (tricarboxylic acid cycle) and antioxidant defense.

### 7.4 Chronic Forced Swim Test (Chronic FST)

#### 7.4.1 Peripheral Material

In addition to acute FST (see ‘Forced swim stress (FST), peripheral material’), Shi *et al.* investigated plasma metabolome alterations in adult male Sprague-Dawley rats not only upon acute FST but also upon chronic FST [93]. In particular, rats were forced to swim for 5 min daily for 14 days. 24h after the last swimming session, rats were sacrificed and plasma samples were used for ^1^H NMR metabolomics analysis. α- and β-glucose, β-hydroxybutyrate, valine and lipids levels were significantly increased in the plasma of the chronic FST compared to the control group, suggesting that chronic FST induced decreased glycogenolysis and glycolysis and increased lipid metabolism, as it was indicated by the elevated glucose levels in the plasma of the chronic FST group compared to controls.

## 8 METABOLOMICS STUDIES IN RODENT MODELS OF EARLY LIFE STRESS

### 8.1 Maternal Separation

#### 8.1.1 Brain Tissue

Cui *et al.* [115] applied a maternal separation protocol in male and female Sprague-Dawley rats by separating the pups from their dam daily from 08:00 to 11:00 and from 14:00 to 17:00, from postnatal day 1 till postnatal day 21, when weaning occurred. Behavioral changes were assessed by SPT, OFT and FST. Maternal separation significantly reduced the body weight and increased anhedonia in both sexes of the stressed animals compared to controls. Immobility time in the FST was significantly lower for the male maternally separated rats compared to controls, while the time spent in the inner zone, the inner zone distance traveled as well as the activity in the OFT were reduced both for male and female maternally separated rats compared to controls, thus highlighting an increased anxiety-related behavior. Whole brain from the maternally separated and non-maternally separated rats was subjected to untargeted metabolomics using ultra-high performance liquid chromatography-triple quadrupole time-of-flight mass spectrometry (UPLC-QTOF-MS). 30 metabolites with altered levels were reported in both sexes of maternally separated mice compared to controls, including glutamine, aspartate, proline, L-glutamic acid 5-phosphate and glutamate that regulate arginine and proline metabolism. Furthermore, increased levels of proline, as well as decreased levels of pantothenic acid and increased levels of pantetheine 4’-phosphate were identified in the maternally separated groups of rats compared to controls. Altered levels of such metabolites may affect CoA synthesis and disturb the ATP synthesis in mitochondria, altering the glutathione levels and oxidative stress, while enhancing depression-like behavior [116, 117].

## 9 COMMON METABOLOMIC PATTERNS IN RODENT MODELS OF STRESS

### 9.1 Current Limitations in Metabolomics Approaches

Based on the aforementioned metabolomics studies in rodent models of stress, several processes/pathways seem to be most prominently affected by stress protocols and are discussed below. However, inherent bias in the metabolomics experimental design, organology used and subsequent *in silico* data analysis should be taken into consideration when interpreting these results. Targeted metabolomics approaches focusing *a priori* on a selected set of metabolites potentially increases detection rates of low abundant metabolites of interest. On the other hand, in untargeted metabolomics approaches, detection and confident quantification of such low abundant metabolites may be masked by high abundant metabolites as well as by the considerable complexity of biological samples such as brain tissue. Furthermore, the varying analytical power of diverse MS and NMR protocols used, the inclusion of annotated metabolites *vs.* not annotated compounds in pathway analysis as well as the overrepresentation of well-characterized biochemical pathways in metabolomics and pathway databases and software may differentially shape metabolomics analysis outcomes.

### 9.2 Amino Acid Metabolism

Affected amino acid biosynthesis/catabolism pathways were observed in most of the included studies in this review regarding acute, chronic and early life stress. In particular, impaired amino acid metabolism was observed in mouse brain and rat plasma upon ARS [83] in several brain regions [97, 98, 101] and plasma [104] of CUMS-exposed rats, in the amygdala of rats subjected to RRS [105] and in the hippocampus of CSDS rats [113]. Additionally, several metabolites associated with amino acid metabolism pathways were found in whole brain metabolome analyses of maternally separated male and female rats [115]. Pathways related to amino acid biosynthesis and catabolism have been implicated in stress responses for several decades [118]. Impaired amino acid metabolism has also been observed in patients suffering from depression with LC-MS analysis [119].

N-acetyl-L-aspartic acid is an amino acid derivative of aspartic acid synthesized in mitochondria and has an emerging role in psychiatry. N-acetyl-L-aspartic acid levels assessed *via* imaging methods is a common marker of bipolar disorder [120]. Systematic analysis of animal models of depression shows that N-acetyl-L-aspartic acid levels are affected in the brains of animal models of stress-induced depression [121]. Moreover, clinical studies support N-acetyl-L-aspartic acid as a potential candidate biomarker of major depressive disorder [122] and an emerging candidate biomarker for post-traumatic stress disorder [123]. N-acetyl-L-aspartic acid levels were found to be affected in the brain of rodent models of stress, using metabolomics as discussed previously. In their study, Fan *et al.* explored metabolome alterations in three distinct brain regions associated with stress and mood regulation, including the amygdala, hippocampus and prefrontal cortex upon CSDS (discussed in the chapter ‘Chronic social defeat stress (CSDS), brain tissue’) [114]. Interestingly, a comparative analysis of identified metabolites in each of the investigated brain-regions showed that both the levels of arachidonic acid and N-acetyl-L-aspartic acid are affected in all brain regions investigated. Specifically, arachidonic acid exhibited the same direction of change in all three brain regions, while N-acetyl-L-aspartic acid was increased in the prefrontal cortex and hippocampus but decreased in the amygdala after CSDS exposure. Additionally, alanine, aspartate and glutamate metabolism were found to be the most affected pathways among the three brain regions [114]. Changes in N-acetyl-L-aspartic acid metabolite levels have also been found in mouse cerebellum upon FST exposure [91], as well as in the study of Liu *et al.*, that investigated brain metabolome alterations upon exposure to either physical or psychological stressors [99]. These data point towards a prominent role of N-acetyl-L-aspartic acid in stress-related effects which warrants further investigation.

### 9.3 Lipid Metabolism

Brain lipids are of major importance for the physiological nervous system functions, as they participate in several fundamental neural processes, including myelination and synaptic neuronal signaling [124]. It has been found that the rat brain lipidome is affected upon CUMS exposure *via* MS-based lipidomics [125]. Lipidomics constitutes a targeted approach to investigate the effects of stress on lipid metabolism, however, the aforementioned metabolomics studies provide useful insights into impaired lipid metabolism in the rat brain and periphery upon chronic stress. In particular, affected lipid metabolism in the hippocampus [98, 99] and the plasma [93] of CUMS rats was observed. In addition, affected glycerophospholipid levels in the hippocampus [96] and serum [103] as well as phospholipid levels in the plasma [104] of rats upon CUMS were found. RRS rats showed altered plasma lipidomic profiles [86] and levels of lipid metabolites in the prefrontal cortex compared to controls [106]. Moreover, CSDS rats had altered lipid metabolite levels in the hippocampus [113] compared to controls. Also, changed levels of lipid metabolites were found in the plasma of rats exposed to 14 days of FST compared to controls [93]. These findings underline the need for multiomics complementary approaches for optimal characterization of stress-pertinent effects.

## 10 PERSPECTIVES

Besides the heterogeneity in the metabolomics workflows used, additional factors such as the organism studied, sex, age, line, the stress protocol (*e.g*., duration, intensity), recovery time and sampling interval after stress exposure may largely influence the metabolomic signatures acquired after stress. Due to the high variability of the identified studies concerning these factors, it is challenging to draw unifying hypotheses and conclusions. Interestingly, in a representative study included in our review, where different stress types were assessed in the same experimental set-up, Liu *et al.* investigated metabolome alterations in the hippocampus of Sprague-Dawley upon 4 different stress-related interventions, including CUMS, learned helplessness, RRS and social defeat [99]. According to the authors, physical (CUMS, learned helplessness) *vs.* psychological (RRS, social defeat) paradigms differentially impacted hippocampal metabolomes. Bioinformatic analysis showed that physical stress affected mainly lipid metabolism and glutamate metabolism, while psychological stress was associated with cell signaling, cellular proliferation and neurodevelopment. Interestingly, the directionality in the level alterations of 9 common metabolites in all groups depended on whether the applied stress paradigm was physical or psychological. These metabolites were mainly associated with sphingosine and sphingosine-1-phosphate metabolism.

### 10.1 Sex-dependent Effects

Most of the identified studies apply chronic stress paradigms to model depression-like behavior using rodent male cohorts, even though depression is more prevalent in women than in men [126]. Therefore, investigating in-depth stress effects in the female brain will help us unravel the contribution of gender in psychopathology and provide us with targeted, gender-specific biomarkers for vulnerable populations. Concerning stress protocols in adult rodents, only one study investigated sex-specific signatures upon stress, namely the levels of 8 steroids, in several brain regions and plasma of Sprague-Dawley rats (discussed in ‘Forced swim stress (FST)') [89]. Plasma corticosterone levels were higher in females in basal and after stress states compared to males and there was not a significant effect of sex in the investigated brain regions in corticosterone levels. Basal progesterone levels in the plasma and basal plasma and brain allopregnanolone levels were higher in females than males. Females, after stress, showed a greater increase in plasma deoxycorticosterone and progesterone levels compared to males. Increased levels of dihydrodeoxycorticosterone were identified only in the frontal cortex of males after stress, while females had elevated dihydrodeoxycorticosterone levels, which were increased in the frontal cortex, hypothalamus, hippocampus and brainstem. In addition, while stress induced elevated dihydroprogesterone levels in all five brain regions analyzed in females, stress increased dihydroprogesterone levels only in the frontal cortex and brainstem in males. Basal plasma and brain allopregnanolone levels were greater in females than males.

Concerning early life stress, Cui *et al.* [115] applied a paradigm of maternal separation on Sprague-Dawley rats and evaluated their behavior in adulthood (see ‘Maternal separation’). In both males and females, depression-like behaviors were assessed, including anhedonia. However, no statistical difference was observed between the sexes. The authors claim that early-life stress models might be unable to replicate gender factor effects, probably due to the lack of rodent sensitivity to the means of behavior measurement or due to the hormonal fluctuation during the estrous cycle of female rodents. Furthermore, the authors claim that the gender factor may not be significant for the occurrence of depression-like behavior in early-life stress models. Further research on male and female rodents is crucial to decipher the molecular and behavioral underpinnings of stress paradigms.

### 10.2 Line-dependent Effects

Basal behavior among different mouse strains differs. In a comparative analysis of C57BL/6 and DBA/2 mice, we found that C57BL/6 mice are characterized by elevated depression-like and reduced anxiety-like behavior compared to DBA/2 mice [127]. Accordingly, the hippocampal and plasma metabolic metabolomic profiles were different between the two mouse strains. Particularly, metabolites implicated in amino acid, nucleotide and mitochondrial metabolism showed level differences between C57BL/6 and DBA/2 mice. Stress differentially affects metabolic and behavioral readouts in a strain-specific manner. O’Mahony *et al.* [128] examined the behavioral and metabolomic parameters of Wistar Kyoto rats and compared them to Sprague-Dawley rats after 2h restraint stress for 10 consecutive days. The Wistar Kyoto showed increased anxiety-related and depression-like behavior compared to the Sprague-Dawley strain, as it was indicated by the FST and OFT, which was also accompanied by stress induced, strain-specific molecular changes.

### 10.3 The Need for Follow-up Studies

A major setback on the current state-of-the-art in the field of stress metabolomics is that despite the quest for following up metabolomics results, a very limited number of studies have performed functional validation of the acquired metabolomics datasets. Among the included acute stress studies, only one followed up their metabolomic results by investigating the upstream pathways that could be related to the increased levels of glutamate and glutamine observed in the prefrontal cortex of ARS mice compared to controls (discussed in ‘Acute restraint stress (ARS), brain tissue’) [83]. In particular, it was found that the levels of a ketone body, β-hydroxybutyrate, and acetyl-CoA were increased in the prefrontal cortex of ARS *vs.* controls using colorimetric assays. As the catabolism of ketone bodies increases acetyl-CoA levels resulting in turn in increased glutamate, glutamine and GABA levels in the brain, the authors investigated and found increased β-hydroxybutyrate-related catabolism enzymes, including pyruvate carboxylase and glutamate dehydrogenase 1, *via* western blot, supporting that ARS induces increased β-hydroxybutyrate metabolism in the prefrontal cortex.

In chronic stress, Linghu *et al.* [100] identified altered levels of several energy-related metabolites (see ‘Chronic unpredictable mild stress (CUMS), brain tissue’) in the hippocampus of CUMS rats *vs.* controls. These results were followed by assessing protein levels and the activity of glucose metabolism-related enzymes as well as mitochondrial microscopy. The CUMS group showed increased activity of glucose metabolism enzymes, namely pyruvate kinase, pyruvate dehydrogenase, pyruvate carboxylase and phosphoenolpyruvate carboxykinase compared to controls, while there were no significant differences in the protein levels of pyruvate kinase, pyruvate dehydrogenase, pyruvate carboxylase and pyruvate carboxylase between the two groups. CUMS also induced increased mitochondrial swelling, cristae breaks and decreased mitochondrial matrix density, ATP levels and mitochondrial membrane potential. CUMS had no effect on mitochondrial respiratory chain complex I, III, and IV enzyme activities. Citrate synthase activity was not affected upon CUMS, yet mitochondrial pyruvate carrier and acetyl-CoA levels were reduced in the CUMS group *vs.* controls. Therefore, the authors suggest that the reduction of mitochondrial pyruvate carrier and acetyl-CoA may be responsible for the reduced ATP synthesis and serve as potential pharmacological targets.

Based on their findings in the prefrontal cortex showing affected nicotinate and nicotinamide metabolism upon CUMS (discussed in ‘Chronic unpredictable stress (CUMS), brain tissue’), Duan *et al.*, [102] followed up their metabolomic results by investigating the protein levels of poly-(ADP-ribose)-polymerase 1 and glutamate receptor 2 precursor that are involved in pathways related to identified affected metabolites. Western blot results revealed that the levels of both proteins were decreased in the prefrontal cortex of the CUMS group compared to controls. In the included RRS studies, Chen *et al.* followed up their metabolomics analysis, which indicated affected plasma histidine metabolism, neurotransmitter synthesis and bile acid metabolism, by treating RRS rats with L-carnosine and observing a significant decrease in the body weight of the treated, stressed group of mice, as well as decreased anxiety-related behavior in the EPM. Such effects were probably mediated *via* the function of L-carnosine on the HPA axis and on the corticosterone secretion [107].

As discussed above (see ‘Chronic social defeat stress ‘CSDS’, brain tissue’), mouse prefrontal cortex metabolome changes upon CSDS exposure included metabolites associated with tryptophan and dopamine metabolism [112]. Towards this direction, the authors also investigated mRNA and protein alterations of tryptophan or dopamine metabolism proteins and found that kynurenine aminotransferases I and III as well as monoamine oxidase A mRNA levels (involved in tryptophan metabolism) were significantly decreased in stress-susceptible mice compared to controls. Moreover, protein levels of glutamate ionotropic receptor NMDA type subunit 2A were increased in stress-susceptible *vs.* control groups and decreased in stress-resilient *vs.* stress-susceptible groups, while glutamate receptor 1 protein levels were increased in the stress-susceptible group and decreased in stress-resilient *vs.* control groups, respectively. Concerning dopamine metabolism, both monoamine oxidase A and catechol-O-methyltransferase mRNA levels as well as dopamine transporter protein levels, were decreased in stress-susceptible *vs.* control animals. Also, dopamine receptor D5 protein levels were increased in the stress-resilient group compared to stress-susceptible and control groups.

Metabolome analysis in the prefrontal cortex of stress-susceptible *vs.* control groups after CSDS included metabolite level alterations associated with tryptophan metabolism, GABAergic and catecholaminergic pathways [111]. To focus on the glutamatergic pathway and follow-up on the metabolomic results, the authors investigated the mRNA and protein levels of key molecules in the glutamatergic pathways and found that mRNA levels of glutamine synthetase were lower in stress-resilient *vs.* control groups. Gad2/Gad65 mRNA levels were both decreased in the stress-susceptible and stress-resilient groups compared to controls. Moreover, Gad1/Gad67 and metabotropic glutamate receptor 1 mRNA levels were decreased in the stress-susceptible group compared to controls. Protein analysis revealed elevated GluA1 levels in the stress-susceptible compared with control mice.

## CONCLUSION

Taken together, combining metabolomics with other -omics technologies and performing integrated data analyses will disentangle molecular stress responses upon different stressors. Follow-up studies of metabolomics outcomes, by pharmacologically targeting enzymes involved in the production of metabolites of interest and/or exploring the genetic technologies available in mice in order to manipulate pertinent pathways, would substantially enrich our understanding of stress-relevant metabolomic signatures and facilitate translational applications for stress-related pathologies.

## Figures and Tables

**Table 1:** Metabolomics studies of acute, chronic and early life rodent models discussed in this review.

**References**	**Acute/ Chronic/ Early Life Stress**	**Stressor**	**Rodent Model**	**Strain**	**Sex**	**Age**	**Behavioral Tests**	**Brain Metabolomics**	**Plasma/Serum ** **Metabolomics**	**Metabolomics Method**
Son *et al*. 2021 [83]	Acute	ARS	Mice	C57BL/6	Male	9 weeks	No	Prefrontal cortex, hippocampus	No	LC-MS/MS
Picard *et al*. 2015 [85]	Acute	ARS	Mice	C57bl/6eij	Male	10-13 months	No	No	Yes	LC-MS
Teague *et al*. 2007 [86]	Acute	ARS	Rats	Sprague-Dawley	Male	n/a	No	No	Yes	NMR
Sze *et al*. 2018 [89]	Acute	FST	Rats	Sprague-Dawley	Male and female	21 weeks	No	Frontal cortex, hypothalamus, hippocampus, amygdala and brainstem	Yes	HPLC-MS
Iliou *et al*. 2021 [91]	Acute	FST	Mice	CD1	Male	7 weeks	FST	Cerebellum	No	NMR
Bassett *et al*. 2019 [92]	Acute	FST	Rats	Wistar Kyoto and Sprague-Dawley	Male	10 weeks	OFT, FST	Whole brain	Yes	HILIC-MS
Shi *et al*. 2013 [93]	Acute	FST	Rats	Sprague-Dawley	Male	n/a	No	No	Yes	NMR
Dulka *et al*. 2017 [94]	Acute	ASDS	Mice	C57BL/6	Male	7-8 weeks	SIT	Basolateral/central amygdala, dorsal hippocampus, nucleus accumbens and ventromedial prefrontal cortex	No	UPLC-HRMS
Geng *et al*. 2020 [95]	Chronic	CUMS	Rats	Sprague-Dawley	Male	8 weeks	SPT, FST	Whole brain	Yes	GC-MS
Geng *et al*. 2019 [96]	Chronic	CUMS	Rats	Sprague-Dawley	Male	8 weeks	SPT, FST	Hippocampus	No	UPLC-MS
Li *et al*. 2021 [97]	Chronic	CUMS	Rats	Sprague-Dawley	Male	n/a	SPT, FST, EPM, OFT	Amygdala	No	LC-MS/MS
Zhang *et al*. 2018 [98]	Chronic	CUMS	Rats	Sprague-Dawley	Male	n/a	SPT, OFT, EPM, FST	Hippocampus	No	GC-MS
Liu *et al*. 2018 [99]	Chronic	CUMS	Rats	Sprague-Dawley	Male	n/a	SPT, FST, OFT, EPM	Hippocampus	No	GC-MS
Ling-Hu *et al*. 2021 [100]	Chronic	CUMS	Rats	Sprague-Dawley	Male	8 weeks	SPT, OFT	Hippocampus	No	LC-MS and stable isotope-resolved metabolomics
Ni *et al*. 2008 [101]	Chronic	CUMS	Rats	Sprague-Dawley	Male	8 weeks	SPT	Cerebral cortex, hippocampus, thalamus, and remaining brain regions	No	GC-MS
Duan *et al*. 2022 [102]	Chronic	CUMS	Rats	Sprague-Dawley	Male	n/a	SPT, FST	Prefrontal cortex	No	LC-MS/MS
Linghu *et al*. 2020 [103]	Chronic	CUMS	Rats	Sprague-Dawley	Male	8 weeks	SPT, OFT	No	Yes	LC-MS and SIRM
Wu *et al*. 2019 [104]	Chronic	CUMS	Rats	Sprague-Dawley	Male	n/a	No	No	Yes	UPLC-MS/MS
Shi *et al*. 2013 [93]	Chronic	CUMS	Rats	Sprague-Dawley	Male	n/a	SPT, OFT	No	Yes	NMR
Li *et al*. 2020 [105]	Chronic	RRS	Rats	Sprague-Dawley	Male	n/a	OFT	Amygdala	No	GC-MS
Liu *et al*. 2016 [106]	Chronic	RRS	Rats	Sprague-Dawley	Male	n/a	SPT, OFT, FST, EPM	Prefrontal cortex	No	GC-MS
Liu *et al*. 2018 [99]	Chronic	RRS	Rats	Sprague-Dawley	Male	n/a	SPT, FST, OFT, EPM	Hippocampus	No	GC-MS
Chen *et al*. 2020 [107]	Chronic	RRS	Rats	Wistar	Male	8 weeks	EPM	No	Yes	UPLC-Q-TOF/MS
Teague *et al*. 2007 [86]	Chronic	RRS	Rats	Sprague-Dawley	Male	n/a	No	No	Yes	NMR
Hamilton *et al*. 2020 [108]	Chronic	CSDS	Mice	C57BL/6 J	Male	7-8 weeks	SIT	Medial prefrontal cortex, nucleus accumbens, ventral hippocampus	Yes	GC-MS and LC-MS/MS
Zhang *et al*. 2020 [110]	Chronic	CSDS	Mice	C57BL/6	Male	7-8 weeks	SIT, TST	Nuclus accumbens, prefrontal cortex, hippocampus	No	GC-MS LC-MS/MS
Wang *et al*. 2016 [111]	Chronic	CSDS	Mice	C57BL/6	Male	2 months	SIT, SPT, OFT	Prefrontal cortex	No	LC-MS/MS
Xu *et al*. 2019 [112]	Chronic	CSDS	Mice	C57BL/6	Male	6-8 weeks	SIT, SPT, OFT	Hippocampus	No	LC-MS/MS
Yang *et al*. 2019 [113]	Chronic	CSDS	Rats	Sprague-Dawley	Male	n/a	SPT, OFT, EPM, FST	Hippocampus	No	GC-MS
Fan *et al*. 2021 [114]	Chronic	CSDS	Rats	Sprague-Dawley	Male	n/a	EPM, SPT, FST, OFT	Amygdala	No	LC-MS
Shi *et al*. 2013 [93]	Chronic	Chronic FST	Rats	Sprague-Dawley	Male	n/a	No	No	Yes	NMR
Cui *et al*. 2020 [115]	Early life	Maternal separation	Rats	Sprague-Dawley	Male and female	63 days	SPT, OFT, FST	Whole brain	No	UPLC-Q-TOF-MS

**Abbreviations:** ARS: acute restraint stress; ASDS: acute social defeat stress; CSDS: chronic social defeat stress; CUMS: chronic unpredictable mild stress; EPM: elevated plus maze; FST: forced swim test; GS-MS: gas chromatography-mass spectrometry; HILIC-MS: hydrophilic interaction chromatography-mass spectrometry; LC-MS/MS: liquid chromatography-tandem mass spectrometry; LC-MS: liquid chromatography-mass spectrometry; NMR: nuclear magnetic resonance; OFT: open-field test; RRS: repeated restraint stress; SIRM: stable isotope-resolved metabolomics; SIT: social interaction test; SPT: sucrose preference test; TST: tail suspension test; UPLC-Q-TOF/MS: ultra-high-performance liquid chromatography-triple quadrupole/time-of-flight mass spectrometry; UPLC-HRMS: ultra-high performance liquid chromatography-high-resolution mass spectrometry; UPLC-MS: ultra-high performance liquid chromatography-mass spectrometry; UPLC-MS/MS: ultra-high performance liquid chromatography-tandem mass spectrometry.

## LIST OF ABBREVIATIONS

ARS: Acute Restraint Stress
ASDS: Acute Social Defeat Stress
CSDS: Chronic Social Defeat Stress
CUMS: Chronic Unpredictable Mild Stress
EPM: Elevated Plus Maze
FST: Forced Swim Test
GS-MS: Gas Chromatography-mass Spectrometry
HPA: Hypothalamic-pituitary-adrenal
HILIC: Hydrophilic Interaction Chromatography
HPLC: High Performance Liquid Chromatography
LC-MS/MS: Liquid Chromatography-tandem Mass Spectrometry
MS: Mass Spectrometry
NMR: Nuclear Magnetic Resonance
OFT: Open-field Test
RRS: Repeated Restraint Stress
SIRM: Stable Isotope-resolved Metabolomics
SIT: Social Interaction Test
SPT: Sucrose Preference Test
TST: Tail Suspension Test
UPLC-HRMS: Ultra-high Performance Liquid Chromatography-high-resolution Mass Spectrometry
UPLC-QTOF-MS: Ultra-high Performance Liquid Chromatography-triple Quadrupole Time-of-flight Mass Spectrometry
